# Structural and Physicochemical Properties of a Chinese Yam Starch–Tea Polyphenol Complex Prepared Using Autoclave-Assisted Pullulanase Treatment

**DOI:** 10.3390/foods12203763

**Published:** 2023-10-13

**Authors:** Sandu Xie, Huiqing Chen, Xinyan Jiang, Bifang Zhou, Zebin Guo, Hongliang Zeng, Yi Zhang

**Affiliations:** 1 School of Life Sciences and Chemistry, Minnan Science and Technology College, Quanzhou 362332, China; xsdfst@163.com (S.X.);; 2 Fujian Provincial Key Laboratory of Quality Science and Processing Technology in Special Starch, Fujian Agriculture and Forestry University, Fuzhou 350002, China; 3 College of Food Science, Fujian Agriculture and Forestry University, Fuzhou 350002, China

**Keywords:** starch–polyphenol complex, physicochemical properties, structural characteristics

## Abstract

Interactions between food components have a positive impact in the field of food science. In this study, the effects of tea polyphenol on the structural and physicochemical properties of Chinese yam starch using autoclave-assisted pullulanase treatment were investigated. X-ray diffraction, Fourier transform infrared spectroscopy, scanning electron microscopy, rapid visco analysis, differential scanning calorimetry, and the 3,5-dinitrosalicylic acid method were applied in this study. The results showed that the Chinese yam starch–tea polyphenol complex formed a structural domain with higher thermal stability along with lower pasting viscosities than native starch. The in vitro digestibility of Chinese yam starch decreased with the addition of the tea polyphenol, and the amount of resistant starch content in the complex was 56.25 ± 1.37%, significantly higher than that of native starch (*p* < 0.05). In addition, the complex showed a B+V-type crystalline structure, which confirmed that the interaction modes between the starch and tea polyphenol include hydrogen bonding and hydrophobic interactions. Moreover, the appearance of an irregular sponge network structure of the complex further supported the interactions between the starch and tea polyphenol. This study provides a theoretical basis for the development of functional foods using Chinese yam starch.

## 1. Introduction

Food products contain complex components such as water, carbohydrates, proteins, and lipids. Different molecular interactions between different food components are ubiquitously present. These interactions usually have dual effects on the quality and functionality of foods, which are hot topics in scientific research circles [1]. The interaction between starch and ligands is an important research topic in the interaction of different components in food. Such ligands are known as complexing agents and include alcohol, lipid, polyphenol, and flavor compounds [2]. Previous studies have shown that the morphology and composition of starch granules are changed by the presence of ligands, and then further affect the gelatinization and retrogradation, resulting in starch with superior properties, such as a stronger water-holding capacity, higher solubility, thermal stability, and lower digestibility [3]. Yam is an important tuber crop grown in tropical and subtropical countries. Yam tubers are rich in protein, vitamins, minerals, and carbohydrates [4], and the carbohydrates of yam tubers are mainly in the form of starch. However, there are few studies focused on the interaction between Chinese yam starch and ligands.

Polyphenols, as natural substances with multiple biological activities, have demonstrated health-promoting properties. The phenolic hydroxyl groups in polyphenols may interact with starch noncovalently, such as through hydrogen bonding, hydrophobic interactions, and electrostatic forces [5]. At the molecular level, there are two types of complexes produced between starch and polyphenols, inclusion complexes and non-inclusive complexes. It is noteworthy that both complexes affect the structural and physicochemical properties of starch to varying degrees [6,7]. In addition, the starch–polyphenol inclusion complex has a crystalline arrangement similar to a starch–lipid inclusion complex classified as type RS5 and can effectively hinder the accessibility to digestive enzymes and reduce the digestibility of starch [8]. On the one hand, it is believed that the resistance of the starch–polyphenol complex results from the formation of a single-helix structure. On the other hand, this resistance may be due to the inhibitory effect of polyphenols on starch digestive enzymes [9,10]. Hence, preparing starch–polyphenol complexes might be more meaningful than starch–lipid complexes overall.

Various studies have demonstrated that the content of amylose is a key factor in forming complexes with ligands, and that amylopectin has a much weaker complexing ability with ligands due to its highly branched structure and steric hindrance [11]. It is worth noting that the high content of amylopectin is a characteristic of Chinese yam starch [12], and it is not beneficial to the interaction between starch and ligands. Pullulanase is a type of starch debranching enzyme that can specifically break the α-1,6 glycosidic bonds at branch points, producing short linear chains, thereby providing binding sites for the interaction between amylose and ligands [13]. However, simple pullulanase action has clear limitations, including a limited amount of short amylose and a narrow complex type [14]. Some studies proposed that a pullulanase combined with physical processing could release more amylose single helices as well as increase the possibility of ligands entering into the starch helical cavity [15]. Generally, autoclave-assisted pullulanase treatment is considered to be a safe, effective, and environmentally friendly method to modify starch. Autoclaving is a method of using pressurized saturated heating steam which has been widely used in the research of starch modification. A previous study showed that compared with native corn starch, starch treated with autoclaving exhibited a complete removal of the amylopectin-ordered structure and further promoted the formation of V-type starch [16]. Li H. et al. [17] reported that the internal structure of starch granules is changed by the autoclaving treatment, resulting in the increased availability of amylose prior to interaction with ligands. In addition, the higher degree of degradation in the starch system induced by autoclave-assisted pullulanase processing offers more arrangement opportunities for short molecular chains [18].

There are no reports in the last decade on autoclave-assisted pullulanase treatment for the preparation of Chinese yam starch–tea polyphenol complexes. Therefore, a Chinese yam starch–tea polyphenol complex was prepared using autoclave-assisted pullulanase treatment, and the effects of tea polyphenol on the structural and physicochemical properties of Chinese yam starch were investigated in this study. The pasting viscosities, thermal properties, in vitro digestibility properties, crystallinity, micromorphology, and the interactions between molecules of the complex were characterized using a variety of modern technologies. The data obtained contribute to providing a theoretical basis for the development of functional foods using Chinese yam starch.

## 2. Materials and Methods

### 2.1. Materials

Chinese yams (Anxi middle lobe variety) were provided by Fujian Shange Agricultural Comprehensive Development Food Co., Ltd. (Quanzhou, China). Based on the results of our previous experiments, the amylopectin content of Chinese yam starch was 69.72 ± 0.499%.

Pullulanase (2000 U/mL, pH 4.5–6.0, liquid, BR) was obtained from Shanghai Macklin Biochemical Co., Ltd. (Shanghai, China). Tea polyphenols [polyphenols 51.16% (UV), catechins 35.7% (HPLC), epigallocatechin gallate 20.8% (HPLC), caffeine 4.90% (HPLC), loss on drying 3.4%] were obtained from Shanghai Titan Technology Co., Ltd. (Shanghai, China), BR. All other chemical reagents used in this study were of analytical grade.

### 2.2. Sample Preparation

#### 2.2.1. Preparation of the Chinese Yam Starch (CYS)

Firstly, the unusable parts of fresh yam tubers had been removed and then mixed with pure water to make a fresh Chinese yam slurry. The fresh Chinese yam slurry was cleaned with two times the volume of pure water and anhydrous ethanol to clarify the top clear liquid. The sediment obtained using this method was the Chinese yam starch. The Chinese yam starch was dried in an oven (101-1AB electric blast drying oven, China) at 40 °C to a constant weight and filtered through a 100-mesh screen.

#### 2.2.2. Preparation of the Modified Starches

The production steps for the Chinese yam starch–tea polyphenol complex (CYS–TPC) were as follows: a Chinese yam starch suspension (5 g of Chinese yam starch, 100 mL of distilled water) was pregelatinized and cooled to 50 °C, and then pullulanase (250 U/g, 1.25 mL) was added to the sample for enzymolysis (65 °C, 20 min). Then, the sample was subjected to a boiling water bath (10 min) to deactivate the enzymes. Tea polyphenol (0.8 mg/mL, 80 mL) was added to the sample and processed in an autoclave (G136D, Zealway, Co., Ltd., Wilmington, NC, USA). The autoclave treatment was carried out at 105 °C for 30 min. Next, the treated sample was refrigerated (4 °C, 24 h) and washed with anhydrous ethanol (repeated three times) to remove uncomplexed tea polyphenol. Then, the sample was freeze-dried (LGJ-12, Xiangyi Laboratory Instrument Development Co., Ltd., Changsha, China) at approximately −80 °C for 24 h before milling and filtering through a 100-mesh screen.

The production steps for the Chinese yam starch (I) were the same as those of the CYS–TPC samples, but pullulanase and tea polyphenol were replaced with equal volumes of distilled water. This sample was named CYS(I).

The production steps for the Chinese yam starch (II) were the same as those of the CYS–TPC samples, but the tea polyphenol was replaced with an equal amount of distilled water. This sample was named CYS(II).

### 2.3. Rapid Visco Analyse Measurements

The viscosity characteristics of native and modified starches during gelatinization were determined using a rapid visco analyzer (Perten Scientific Instruments Ltd., Stockholm, Sweden). A sample suspension of 8% was prepared and equilibrated at room temperature for 24 h. The test conditions were as follows: Firstly, the suspension was held at 50 °C for 1 min, heated to 95 °C within 5 min, held at 95 °C for 11 min, cooled to 50 °C within 4 min, and subsequently held at 50 °C for 3 min. The paddle speed was 960 rpm during the first 10 s to disperse the sample, and then the paddle speed was set at 160 rpm during the measurement.

### 2.4. Thermal Properties Measurements

The energy change in the samples during gelatinization was measured via differential scanning calorimetry (DSC-200 F3, NETZSCH, Selb, Germany). The sample and purified water were added to the liquid crucible at a ratio of 1:2, and then left to equilibrate at room temperature for 24 h. The test conditions were as follows: the equipment was heated from 20 °C to 120 °C at a speed of 20 °C/min, and the onset, peak, and completion temperatures were obtained. Afterward, the gelatinization enthalpy was calculated according to the peak area.

### 2.5. In Vitro Digestion Measurements

**Figure 1:**
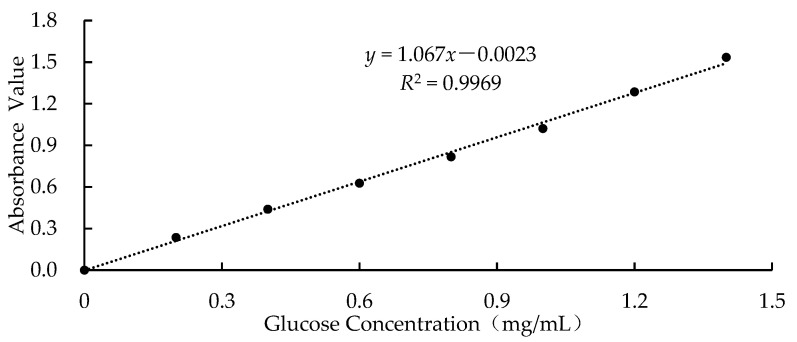
Standard curve of the glucose.

The measurement method for the content of the free glucose in the starch was as follows [19]: the starch sample (0.3 g) was added to distilled water (5 mL), stirred well to combine, and then incubated at 50 °C in a shaking water bath at 150 rpm for 20 min. Finally, the mixed solution was centrifuged at 3500 rpm for 10 min, and 10 mL of the supernatant was used to measure the glucose content using the 3,5-dinitrosalicylic acid (DNS) method. In addition, the amount of free glucose in the sample was calculated according to the equation in Figure 1.

The in vitro digestibility of the native and modified starches was determined based on the method described by Khurshida et al. [20] with a slight modification. A starch sample (300 mg) was added to a 0.2 M phosphoric acid buffer (20 mL, pH 5.2). The enzyme solution was prepared using α-amylase (9 U/mg, Yuanye Biotechnology) and glucoamylase (100,000 U/g, Yuanye Biotechnology) dispersed in distilled water (15 mL). The above materials were transferred to a dialysis bag, and then incubated at 37 °C in a shaking water bath at 150 rpm. Aliquots (1 mL) of the hydrolyzed solution were taken after 20 min and 120 min of incubation and mixed with 95% ethanol (3 mL) to inactivate the enzymes. The mixed solution was centrifuged at 3000 rpm for 10 min, and the supernatant of the mixed solution was used to measure the glucose content using the 3,5-dinitrosalicylic acid (DNS) method. The rapidly digestible starch (*RDS*) and slowly digestible starch (*SDS*) were measured after incubation for 20 min and 120 min, respectively. The resistant starch (*RS*) was the undigested starch after 120 min of incubation. The hydrolysis rate (*HR*, %) of starches and the content of *RDS*, *SDS*, and *RS* in the starch were calculated according to the following equations:

(1) $$G(t)(mg)=\frac{G}{V}\times (331-V)+A-FG$$

(2) $$HR(\%)=\frac{G(t)}{W}\times 100$$

(3) $$SDS(\%)=\frac{\left[G(120)-G(20)+FG\right]\times 0.9}{W}\times 100$$

(4) $$RDS(\%)=\frac{G(20)\times 0.9}{W}\times 100$$

(5) RS(%)=100−RSD−SDS

where *G*(*t*) is the amount of glucose in the starch sample after *t* min of digestion, mg; *G* is the amount of glucose in the standard curve, mg; *V* is the volume of solution removed from the digestion system, mL; 331 is the total volume of the digestion system, mL; *A* is the amount of glucose contained in the solution removed from the digestion system, mg; *FG* is the amount of free glucose contained in the original starch sample, mg; 0.9 is the conversion factor of starch and glucose; *W* is the amount of the starch sample on a dry basis; and *t* is the response time, h.

### 2.6. X-ray Diffraction (XRD) Spectral Measurement

A smartLab X-ray diffractometer (Rigaku Corporation, Tokyo, Japan) was used to determine the crystallinity of the sample powder. The operating parameters used were as follows: the target was Cu-Kα radiation, and the scanning range was 3–40° with a scanning speed of 5°/min.

### 2.7. Fourier Transform Infrared (FT-IR) Spectral Measurement

The vibrational spectra of the samples were determined using a Perkin Elmer FTIR spectrometer (IR Affinity-1S, Shimadzu Enterprise Management, Shanghai, China). The spectra were recorded with 25 scans in the wavenumber range 400–4000 cm^–1^ with 4 cm^−1^ resolution at room temperature (about 25 °C) using a diffuse reflectance accessory. Samples were diluted with KBr (1:100 *v*/*v*) before acquisition, and the background value from pure KBr was acquired before the samples were scanned.

### 2.8. Scanning Electron Microscopy (SEM)

The morphologies of the starch granules were observed at 1000× and 10,000× magnifications using an SEM (MERLIN Compact-61-73, Zeiss, Oberkochen, Germany). The powder was fixed to the SEM stub with double-sided tape. The floating powder was blown off, and the remaining powder was sprayed twice with gold. The entire operation was performed under vacuum conditions (1.81 × 10^−6^ mbar) with an accelerating voltage of 3.00 keV.

### 2.9. Statistical Analysis

All measurements were made in triplicate, and the graphic processing of all experiments was performed using Origin Pro 8.5. The data were analyzed, and significant differences were determined using DPS 9.05 (Science Press, Beijing, China). *p* ≤ 0.05 was considered statistically significant.

## 3. Results and Discussion

### 3.1. Pasting Properties

The pasting properties of native and modified starches are listed in Table 1. The PV reflects the degree of expansion of starch granules during the gelatinization process. The BD reflects the stability of the starch paste, which is defined as the difference between the PV and TV. The smaller BD reflects the higher stability of starch granules and indicates they were difficult to destroy [21]. In addition, the SB is defined as the difference between the FV and TV, which reflects the stability of the starch paste during retrogradation. A higher SB demonstrates that the short-term retrogradation of starch was enhanced [22]. Compared with CYS, the BD and SB of modified starches were all significantly decreased, which indicates that they were more tolerant to shear and heating, and thermal stability was improved [23]. Due to multiple gelatinization treatments, the double helix structure of CYS(I) was destroyed and the granule structure loosened, resulting in a weaker swelling capacity. CYS(II) underwent pullulanase-mediated debranching on the basis of CYS(I), and its double helix structure and long amylopectin were further damaged [24], which effectively reduced the steric hindrance between molecules, resulting in decreasing pasting viscosities. The pasting properties of CYS–TPC were the lowest among all the starch samples. This phenomenon might be related to the numerous hydroxyl groups present in the tea polyphenol that formed hydrogen bonds with amylose and competed with starch for the opportunity to combine with water molecules [25]. This hindered the leaching of amylose, and the swelling of the starch granules weakened, which reduced the PV, TV, and FV of CYS–TPC. The SB of CYS–TPC was the lowest, indicating that the retrogradation of starch was significantly attenuated by the addition of tea polyphenol. This might be ascribed to the fact that a stable inclusion structure formed between the tea polyphenol and starch during autoclaving, which hindered the reassociation of amylose molecules and effectively impeded the retrogradation process [26].

### 3.2. Thermal Properties

**Figure 2:**
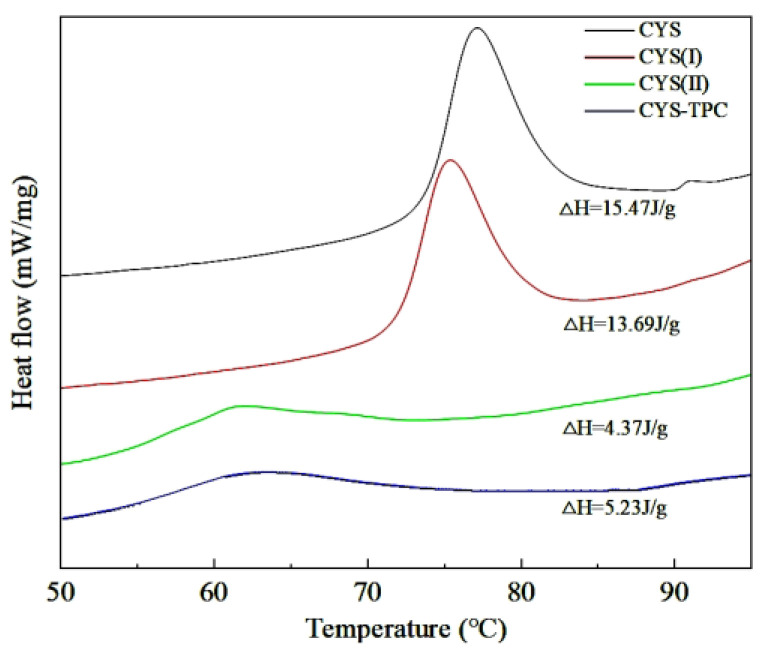
DSC curves of native and modified starches. The abbreviations CYS–TPC, CYS(II), CYS(I), CYS represent, separately, Chinese yam starch–tea polyphenol complex, Chinese yam starch (II), Chinese yam starch (I), and Chinese yam starch.

A significant variation in thermal properties between native and modified starches was obtained, as shown in Figure 2 and Table 2. CYS showed a phase transition in the range of 74.21–82.05 °C with a Δ*H* of 15.47 J/g. The modified starches exhibited a lower *T*o, *T*p, *T*c, Δ*T*, and Δ*H* value in comparison with CYS. A high Δ*T* value has been related to a high degree of crystallinity, which produces structural stability and causes the starch granules to be more resistant to gelatinization. The Δ*H* value mainly reflects the energy required to break the double helix structure of starch during gelatinization [27]. The order of the Δ*T* value of the four samples was CYS–TPC > CYS(II) > CYS > CYS(I), which made clear that the relative crystallinity of CYS–TPC was the highest, indicating that CYS–TPC did not gelatinize easily and was more resistant to enzymatic hydrolysis. Compared to CYS, the lower Δ*H* value of the modified starches might be because of their slack amylopectin double helices, and, thus, less enthalpy was required for their pasting process [28]. In contrast, the Δ*H* value of CYS(II) was significantly lower than that of CYS(I), which was probably due to the further destruction of the double helix structure of amylopectin during pullulanase treatment [29]. The Δ*H* value of CYS–TPC was slightly higher than CYS(II), indicating the interaction between starch and tea polyphenol stabilized the double or single helix structure, thus enhancing thermal stability [30]. This might be attributed to the autoclaving process, which may have induced a new and stronger interaction between the tea polyphenol and amylose chains and reinforced the crystalline structure of CYS–TPC. This then led to higher temperatures required to dissociate the stable structure. Furthermore, the tea polyphenol increased the steric hindrance to limit the leaching of amylose and inhibit swelling of Chinese yam starch, thus increasing the value of Δ*H* [31].

### 3.3. In Vitro Digestive Properties

The in vitro digestive properties of the native and modified starches are presented in Table 3. The digestibility of starch depends on the sensitivity of the starch to enzymes, and the sensitivity is closely related to the microstructure of the starch, including granule surface morphology, amylopectin chain length, amylose concentration, particle size, and crystallinity [32]. The RS contents of CYS(I) and CYS(II) were lower than that of CYS, whereas the RS content of CYS–TPC was higher. The reason for the decreased fraction of RS in CYS(I) might be due to the disrupted structure of starch granules during the process of repeated heating, which caused the starch to be easily broken down by digestive enzymes [33]. For this reason, the hydrolysis rate (*HR*, %) of CYS(I) at 20 min and 120 min was also significantly higher than that of CYS. The RS content of CYS(II) slightly increased compared to that of CYS(I). The reason might be related to the rearrangement of short amylose produced after pullulanase treatment. The broken starch chains realigned with the amorphous region, which reduced the number of attack sites for enzymes, enhancing the resistance to digestion and contributing to a greater RS content [34]. However, there was no significant difference between the CYS(I) and CYS(II) hydrolysis rate (*HR*, %) at 20 min and 120 min. The resulting hydrolysis rate (*HR*, %) might have been related to the treatment conditions or the composition of starch and might also depend on the starch molecular chain length distribution, which should be further investigated. CYS–TPC showed the highest RS content (56.25 ± 1.37%), whereas the lowest *RDS* content (22.92 ± 0.14%) illustrated that tea polyphenol effectively resisted the digestion of starch. Furthermore, the hydrolysis rate (*HR*, %) of CYS–TPC at 120 min was 45.08 ± 1.72%, lower than that of the other starches. This result might be attributed to the interaction between the starch and tea polyphenol, which led to a higher degree of aggregation of the starch molecules, Making it difficult for enzymes and water to enter the starch granules. Furthermore, tea polyphenol may bind with the active sites of the starch digestion enzymes, causing competitive inhibition [35].

### 3.4. X-ray Diffraction Properties

**Figure 3:**
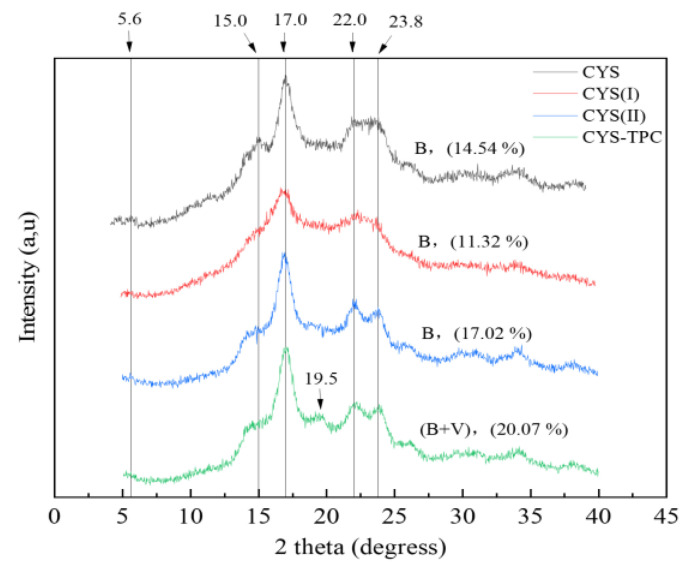
X-ray diffraction patterns of native and modified starches. The abbreviations CYS–TPC, CYS(II), CYS(I), and CYS represent, separately, Chinese yam starch–tea polyphenol complex, Chinese yam starch (II), Chinese yam starch (I), and Chinese yam starch.

The XRD patterns and relative crystallinity of the native and modified starches are shown in Figure 3. CYS showed apparent peaks at 5.6°, 15.0°, 17.0°, 22.0°, and 23.8°, corresponding to a B-type crystalline structure. No significant change in the position of the diffraction peaks was observed for CYS(I) or CYS(II), implying that the autoclave-assisted pullulanase treatment had no influence on the crystalline pattern of Chinese yam starch, and, thus, the two samples exhibited B-type crystalline structures. However, CYS(I) showed a decrease in relative crystallinity from 14.54% to 11.32% compared with the native starch. This result showed the crystalline regions of starch or the hydrogen bonds between double helix granules had been destroyed during the pasting process. In contrast, the short linear chain content in CYS(II) increased after pullulanase treatment, which led to higher molecular mobility and promoted starch recrystallization at a low temperature, resulting in a relative crystallinity increase of 17.02%. With the addition of tea polyphenol during autoclaving, CYS–TPC showed an apparent peak at 19.5°, which was a typical peak of a V-type crystalline structure, and, therefore, CYS–TPC showed a B+V-type crystalline structure. This result demonstrated that stable clusters might be formed between tea polyphenol and the amylose helical cavity through hydrophobic interactions. At the same time, a non-inclusive structure formed between starch side chains, and tea polyphenol was weakly linked by hydrogen bonds [36]. Moreover, compared with other starches, the relative crystallinity of CYS–TPC increased to 20.07%, which indicated that CYS–TPC had more crystalline regions and a more compact structure and, thus, exhibited more resistant to enzymatic degradation [37].

### 3.5. FT-IR Spectroscopy Properties

**Figure 4:**
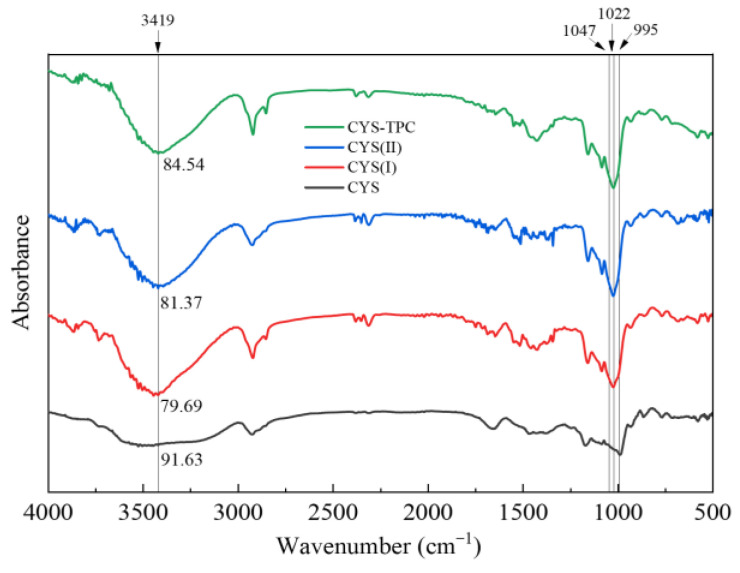
FTIR spectra of native and modified starches. The abbreviations CYS–TPC, CYS(II), CYS(I), and CYS represent, separately, Chinese yam starch–tea polyphenol complex, Chinese yam starch (II), Chinese yam starch (I), and Chinese yam starch.

The FT-IR spectra of native and modified starches are presented in Figure 4. All samples presented similar and typical FT-IR spectrograms. There were no disappearances or appearances of new peaks in the modified starches, which indicated that the modified technique in this study produced physical but not chemical changes in the starches. The IR bands obtained between 3000 and 3600 cm^−1^ were associated with O-H stretching and hydrogen bonds. The weaker intensity of the absorption peak at 3419 cm^−1^ reflected a lower content of O-H and higher content of hydrogen bonds [38]. Compared with CYS, the absorption peak of the O-H bending vibration of the modified starches was significantly narrowed. The order of peak sizes was CYS > CYS–TPC > CYS(II) > CYS(I), which reflected the higher content of hydrogen bonds formed in the starch molecules during the modification process. This result suggested a pronounced effect of the modified treatment used in this study on starch degradation and molecular rearrangement during recrystallization and that the effect of adding tea polyphenol during autoclaving was very significant. It could also be inferred that the tea polyphenol interacted with starch mainly via hydrogen bonding, and the hydrogen bond forces formed in CYS–TPC were stronger than those between starch molecules [39].

The ratio between 1047 cm^−1^ and 1022 cm^−1^ (*R*_1047/1022_) and the ratio between 1022 cm^−1^ and 995 cm^−1^ (*R*_1022/995_) of native and modified starches are summarized in Table 4. A greater value of *R*_1047/1022_ reflects a higher starch crystallinity. A smaller value of *R*_1022/995_ reflects a more ordered double helix structure of the starch [40]. Compared with CYS, the *R*_1047/1022_ values of the modified starches increased significantly, and the highest *R*_1047/1022_ value was in CYS–TPC, indicating that CYS–TPC had a more ordered crystalline structure. In particular, the *R*_1047/1022_ values of native and modified starches were ordered as CYS–TPC > CYS(II) > CYS(I) > CYS, which was consistent with the conclusion of the XRD analysis. Except for CYS(I), the *R*_1022/995_ values of CYS(II) and CYS–TPC were significantly lower than that of CYS, indicating that the double helix structure of CYS(I) was destroyed by high temperature, but the effect of the pullulanase and tea polyphenol might play a positive role in enhancing the double helix structure [41]. Furthermore, the *R*_1022/995_ value of CYS–TPC was lower than that of CYS(II), demonstrating that a more perfect crystalline structure formed with the interaction of tea polyphenols and starch.

### 3.6. Morphological Properties

**Figure 5:**
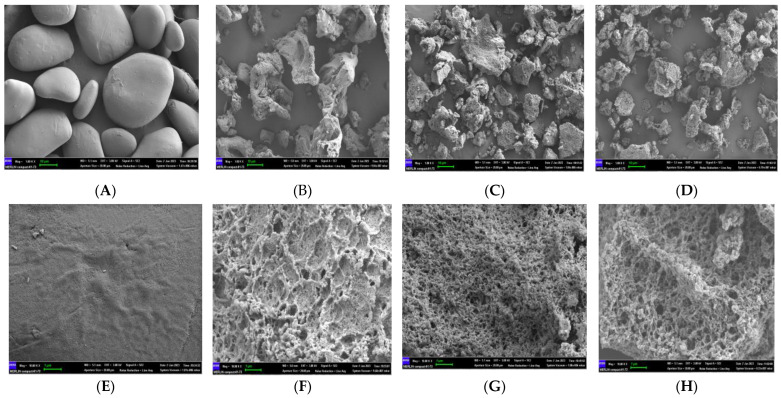
Scanning electron micrograph (SEM) images (1000×) of CYS (**A**), CYS(I) (**B**), CYS(II) (**C**), and CYS–TPC (**D**) and scanning electron micrograph (SEM) images (10,000×) of CYS (**E**), CYS(I) (**F**), CYS(II) (**G**), and CYS–TPC (**H**). The abbreviations CYS–TPC, CYS(II), CYS(I), and CYS represent, separately, Chinese yam starch–tea polyphenol complex, Chinese yam starch (II), Chinese yam starch (I), and Chinese yam starch.

The morphological observation of native and modified starch granules scanned via SEM is shown in Figure 5. The CYS granules had an irregular oval shape, with a smooth and flat surface. In contrast, the modified starch granules were observed to have irregular shapes, rough surfaces, and obvious pores. The size and shape of the pores in the modified starch granules as determined using different technologies were significantly different. A very twisted shape appeared on the CYS(I) granules (Figure 5B), whereas the CYS(II) and CYS–TPC granules displayed uniformly sized pores (Figure 5C,D). CYS(II) granules exhibited a dense and ordered three-dimensional network and had relatively smaller pores and thicker pore walls compared to CYS(I) granules (Figure 5F,G). This suggested that Chinese yam starch might be sensitive to pullulanase and that the short amylose produced by pullulanase effectively increased the cross-linking of starch granules [42]. However, the CYS–TPC granules exhibited a hollow structure with more porous holes and a relatively fluffy structure, which verified a destructive effect on the starch gel network structure with the addition of tea polyphenol during autoclaving [43].

## 4. Conclusions

This study provides a theoretical basis for further research on the functional characteristics of the Chinese yam starch–tea polyphenol complex as a resistant starch. This study investigated the influences of tea polyphenol on pasting, thermal, in vitro digestive, and structural properties of Chinese yam starch using autoclave-assisted pullulanase processing. Tea polyphenol exhibited an inhibitory effect on the viscoelasticity of Chinese yam starch but had a positive role in enhancing thermal stability. Furthermore, tea polyphenols might interact with starch through non-covalent interaction, such as hydrogen bonds and hydrophobic interactions, according to the Fourier infrared spectroscopy and X-ray diffraction results. In particular, hydrogen bonding was speculated to be the main force of interaction between Chinese yam starch and tea polyphenol, which led to CYS–TPC presenting a B+V-type crystalline structure. Furthermore, the number of the RS fractions in the resulting CYS–TPC was effectively enhanced through the addition of tea polyphenol, causing the complex to show a lower digestion efficiency. In terms of its microscopic morphology, CYS–TPC was observed to be more porous with a looser microstructure and irregular cavities, which revealed that the starch gel structure was destroyed by the presence of tea polyphenol. However, the CYS-TPC in the study underwent several gelatinization treatments during preparation, which might significantly affect the physicochemical and structural properties of the CYS-TPC. Based on this situation, the degree of gelatinization in the physicochemical and structural properties of CYS-TPC should be further explored to supplement the shortcoming of this study. Additionally, in order to further improve the research content, future work is required to investigate the edible safety of CYS-TPC and the biological activity function of tea polyphenols within the complex.

## Figures and Tables

**Table 1:** Pasting properties of native and modified starches.

Samples	PV (mPa·s)	TV (mPa·s)	BD (mPa·s)	FV (mPa·s)	SB (mPa·s)	PT (°C)
CYS–TPC	976.03 ± 2.89 ^d^	921.27 ± 1.93 ^c^	55.37 ± 0.67 ^d^	1024.27 ± 1.37 ^d^	103.23 ± 1.06 ^d^	72.63 ± 1.28 ^c^
CYS(II)	995.40 ± 2.79 ^c^	923.13 ± 1.82 ^c^	72.40 ± 0.92 ^c^	1090.17 ± 1.63 ^c^	167.13 ± 1.65 ^c^	69.58 ± 1.12 ^d^
CYS(I)	1080.42 ± 4.11 ^b^	987.37 ± 2.19 ^b^	93.53 ± 0.95 ^b^	1176.17 ± 1.46 ^b^	189.15 ± 1.16 ^b^	74.82 ± 1.03 ^b^
CYS	1197.20 ± 2.88 ^a^	1069.33 ± 3.07 ^a^	128.33 ± 1.34 ^a^	1579.07 ± 1.55 ^a^	510.47 ± 1.91 ^a^	81.94 ± 0.99 ^a^

The abbreviations CYS–TPC, CYS(II), CYS(I), and CYS represent, separately, Chinese yam starch–tea polyphenol complex, Chinese yam starch (II), Chinese yam starch (I), and Chinese yam starch. Values are the means of three replicates ± SD. Values with different superscripts in the same column differ significantly (*p* < 0.05). PV, peak viscosity; TV, trough viscosity; BD, breakdown viscosity; FV, final viscosity; SB, set back viscosity; PT, pasting temperature.

**Table 2:** Thermal properties of native and modified starches.

Samples	*T*o (°C)	*T*p (°C)	*T*c (°C)	Δ*H* (J/g)	Δ*T* (°C)
CYS–TPC	55.62 ± 0.95 ^c^	62.45 ± 0.75 ^c^	73.31 ± 0.51 ^c^	5.23 ± 0.19 ^c^	17.69 ± 0.75 ^a^
CYS(II)	54.23 ± 1.11 ^c^	61.37 ± 0.98 ^c^	69.23 ± 0.88 ^d^	4.37 ± 0.21 ^d^	15.01 ± 0.46 ^b^
CYS(I)	70.31 ± 0.48 ^b^	71.55 ± 0.72 ^b^	75.22 ± 0.62 ^b^	13.69 ± 0.12 ^b^	4.91 ± 0.52 ^d^
CYS	74.21 ± 0.72 ^a^	77.37 ± 0.54 ^a^	82.05 ± 0.35 ^a^	15.47 ± 0.13 ^a^	7.84 ± 0.54 ^c^

The abbreviations CYS–TPC, CYS(II), CYS(I), and CYS represent, separately, Chinese yam starch–tea polyphenol complex, Chinese yam starch (II), Chinese yam starch (I), and Chinese yam starch. Values are the means of three replicates ± SD. Values with different superscripts in the same column differ significantly (*p* < 0.05). *T*o, Onset temperature; *T*p, Peak temperature; *T*c, Conclusion temperature; Δ*H*, Enthalpy of gelatinization; Δ*T* = *T*c – *T*o, gelatinization range.

**Table 3:** The in vitro digestive properties of native and modified starches.

Samples	*HR* (%)		*RDS* (%)	*SDS* (%)	*RS* (%)
	20 min	120 min			
CYS–TPC	25.47 ± 0.16 ^c^	45.08 ± 1.72 ^c^	22.92 ± 0.14 ^c^	22.50 ± 1.65 ^b^	56.25 ± 1.37 ^a^
CYS(II)	33.23 ± 0.73 ^a^	58.36 ± 0.26 ^a^	29.90 ± 0.66 ^a^	23.05 ± 0.64 ^b^	47.05 ± 0.23 ^c^
CYS(I)	34.36 ± 0.51 ^a^	58.22 ± 0.47 ^a^	30.93 ± 0.46 ^a^	27.28 ± 0.81 ^a^	41.79 ± 0.43 ^d^
CYS	29.47 ± 1.05 ^b^	54.74 ± 0.52 ^b^	26.52 ± 0.94 ^b^	22.95 ± 1.40 ^b^	50.53 ± 0.47 ^b^

The abbreviations CYS–TPC, CYS(II), CYS(I), and CYS represent, separately, Chinese yam starch–tea polyphenol complex, Chinese yam starch (II), Chinese yam starch (I), and Chinese yam starch. Values are the means of three replicates ± SD. Values with different superscripts in the same column differ significantly (*p* < 0.05). *HR*, hydrolysis rate; *RDS*, rapidly digestible starch; *SDS*, slowly digestible starch; *RS*, resistant starch.

**Table 4:** Order and double helix degree of starch samples.

Samples	995 cm^−1^	1022 cm^−1^	1047 cm^−1^	*R*_1047/1022_	*R*_1022/995_
CYS–TPC	79.5296 ± 0.5191 ^c^	75.7965 ± 0.1769 ^d^	79.9586 ± 0.2591 ^d^	1.0549 ± 0.003017 ^a^	0.9531 ± 0.005615 ^b^
CYS(II)	83.8199 ± 0.5869 ^b^	79.4661 ± 0.09897 ^c^	83.4058 ± 0.1702 ^c^	1.0496 ± 0.001282 ^b^	0.9481 ± 0.005579 ^b^
CYS(I)	83.1542 ± 0.4042 ^b^	83.9323 ± 0.1030 ^b^	84.1725 ± 0.1841 ^b^	1.0029 ± 0.001713 ^d^	1.0094 ± 0.004023 ^a^
CYS	89.8651 ± 0.3591 ^a^	90.6462 ± 0.3455 ^a^	91.7232 ± 0.2093 ^d^	1.0119 ± 0.001598 ^c^	1.0087 ± 0.0009833 ^a^

The abbreviations CYS–TPC, CYS(II), CYS(I), and CYS represent, separately, Chinese yam starch–tea polyphenol complex, Chinese yam starch (II), Chinese yam starch (I), and Chinese yam starch. Values are the means of three replicates ± SD. Values with different superscripts in the same column differ significantly (*p* < 0.05). *R*_1047/1022_, the ratio between 1047 cm^−1^ and 1022 cm^−1^; *R*_1022/995_, the ratio between 1022 cm^−1^ and 995 cm^−1^.

## Data Availability

The data used to support the findings of this study can be made available by the corresponding author upon request.
